# THE RELATIONSHIP BETWEEN STAFFING AND QUALITY FOR CARE HOME RESIDENTS: A REALIST REVIEW

**DOI:** 10.1093/geroni/igz038.1396

**Published:** 2019-11-08

**Authors:** Karen Spilsbury, Kirsty Haunch

**Affiliations:** 1 University of Leeds, Leeds, United Kingdom; 2 University of Leeds, Leeds, England, United Kingdom

## Abstract

In recent years, quality of care in the sector has come under increasing scrutiny, and practice and policy concerns have been raised about staffing levels, recruitment and retention. Beyond broad recognition that ‘staff influence quality’, little is known about the care home workforce and its relationship to quality. We are undertaking the first study in the UK to address this important question, considering quality from a range of perspectives. One work package comprises a realist review to develop theoretical explanations of how, why and in what circumstances staffing promotes quality for residents and relatives. We will present three mechanisms that promote quality - individual commitment to resident focused care, reciprocity in the team, and a mandate to be creative and flexible to meet residents’ needs. We will consider the contexts required to trigger these mechanisms – staffing numbers, stability of the team, size of the team, and clear leadership.

